# Supplementary material for: Nutrient conditions affect antimicrobial pharmacodynamics in Pseudomonas aeruginosa
Source: Microbiol Spectr. 2024 Dec 10;13(1):e01409-24. doi: 10.1128/spectrum.01409-24 (PMC11705865; doi:10.1128/spectrum.01409-24)
Supplement: Supplemental material — Figures S1 to S6; Tables S1 to S3. [file spectrum.01409-24-s0001.pdf]

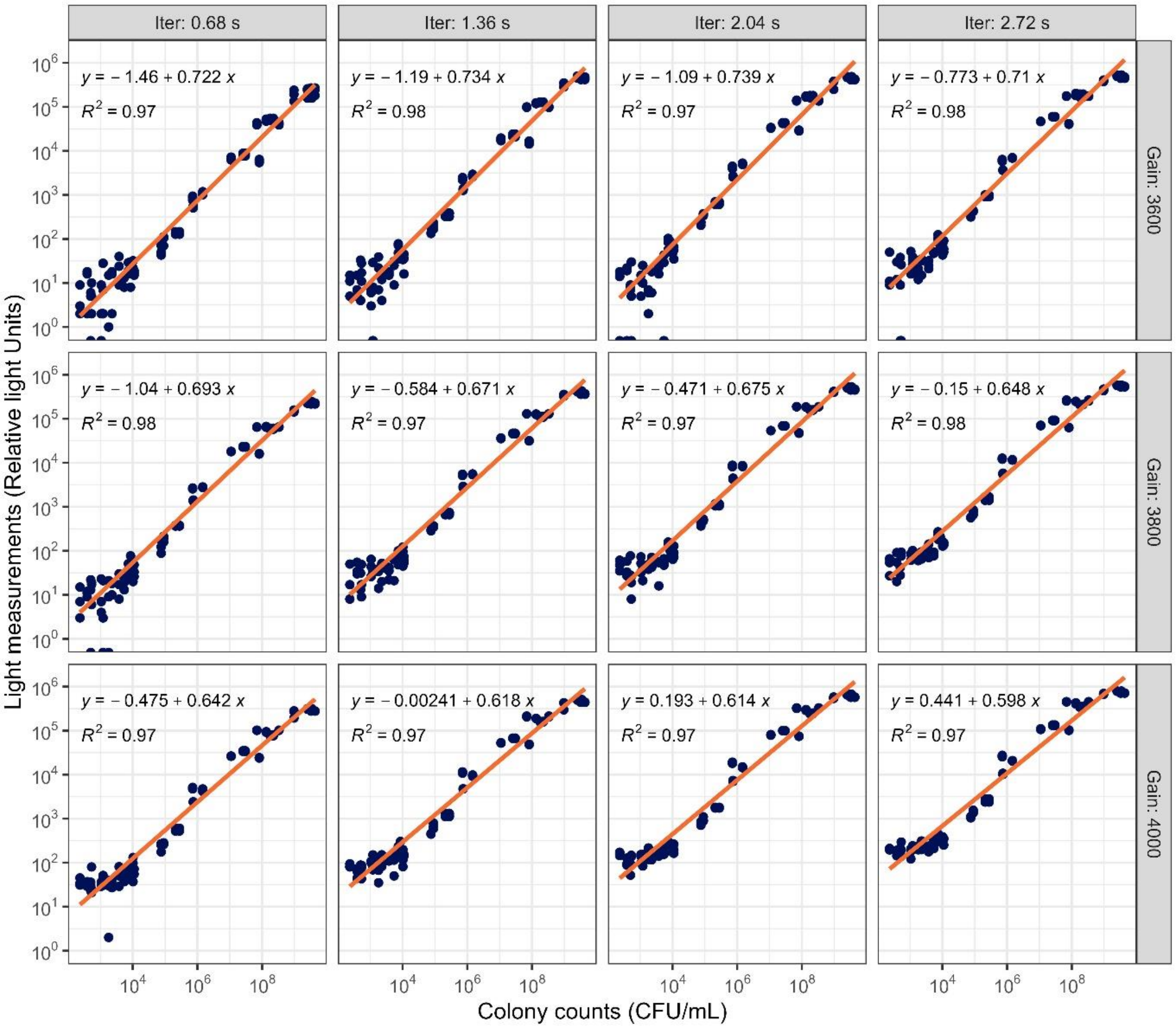

Supplemental Figure 1. Linear calibration between luminescence (relative light units, RLU) and cell counts (CFU/mL) for multiple combinations of detector settings, varying iteration time (iter, columns) and gain (rows). The iteration time stands for the total measurement time per well and the gain is amplification in the conversion from light into electric signal.

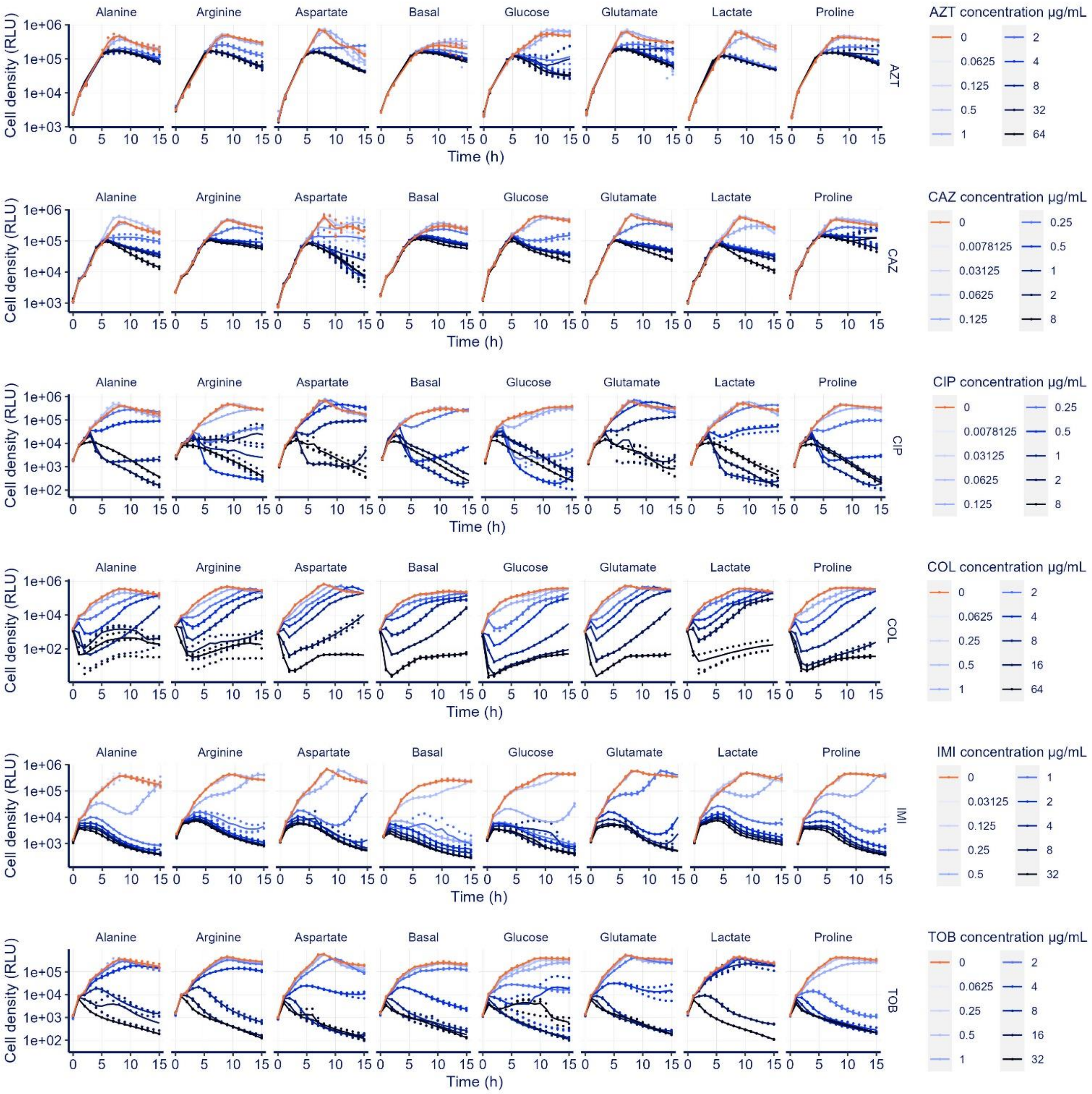

Supplemental figure 2. *Dynamic analysis of the population size over time during the treatment of 6 antibiotics with 9 concentrations and a positive control in 8 media formulations. The y-axis is the cell density measured by relative light units (RLU). All conditions have 3 biological replicates. Abbreviations: aztreonam (AZT), ceftazidime (CAZ), ciprofloxacin (CIP), colistin (COL), imipenem (IMI), and tobramycin (TOB).*

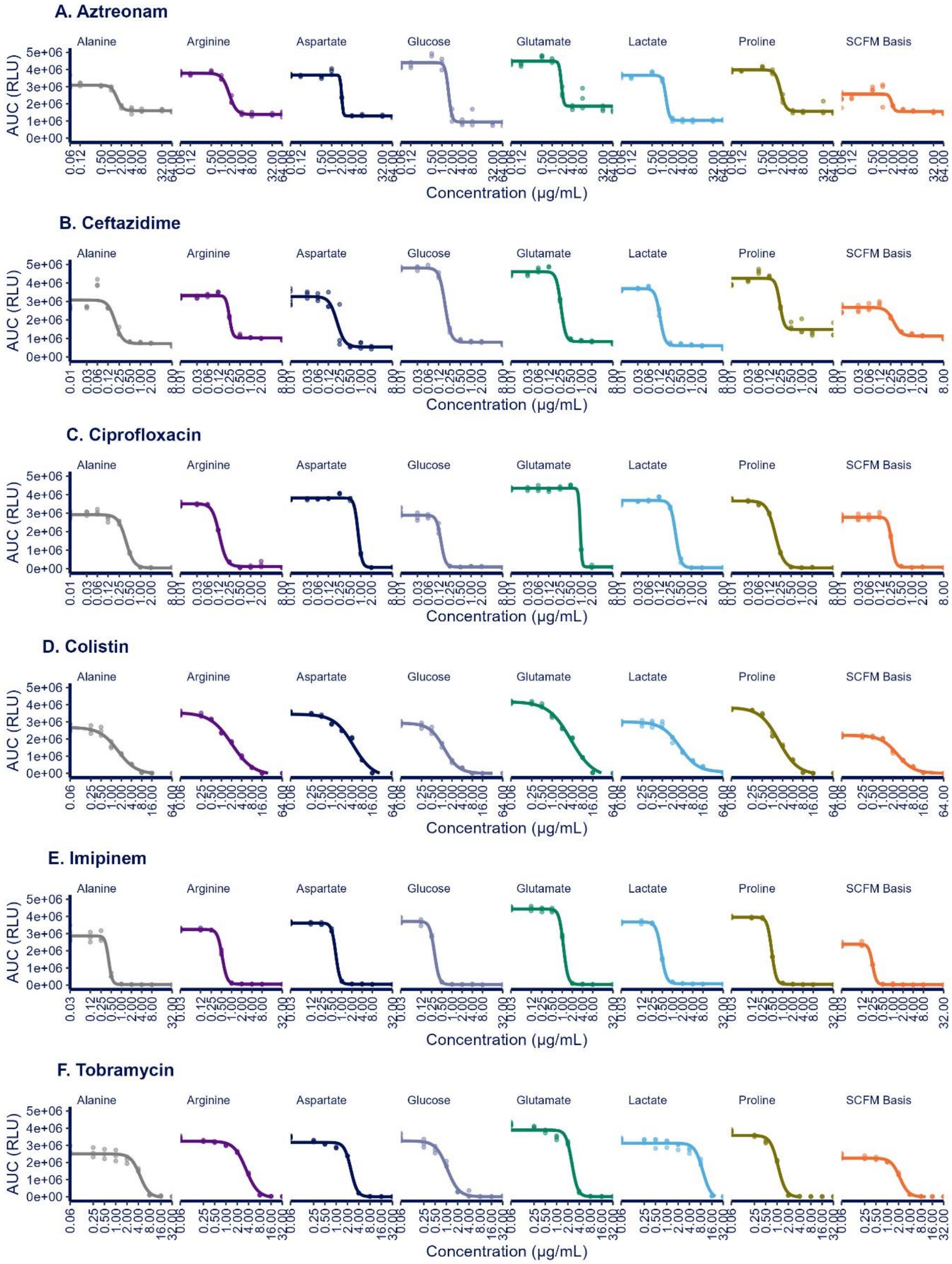

Supplemental Figure 3. Emax model fitting was performed on the area under the curve (AUC) of growth curves across varying antibiotic concentrations. The model was fitted using the average AUC values for each antibiotic concentration (n = 3). From this model, the upper limit (E_0), the half-maximal effective concentration (EC_{50}), and the lower limit (E_{max}) were determined.

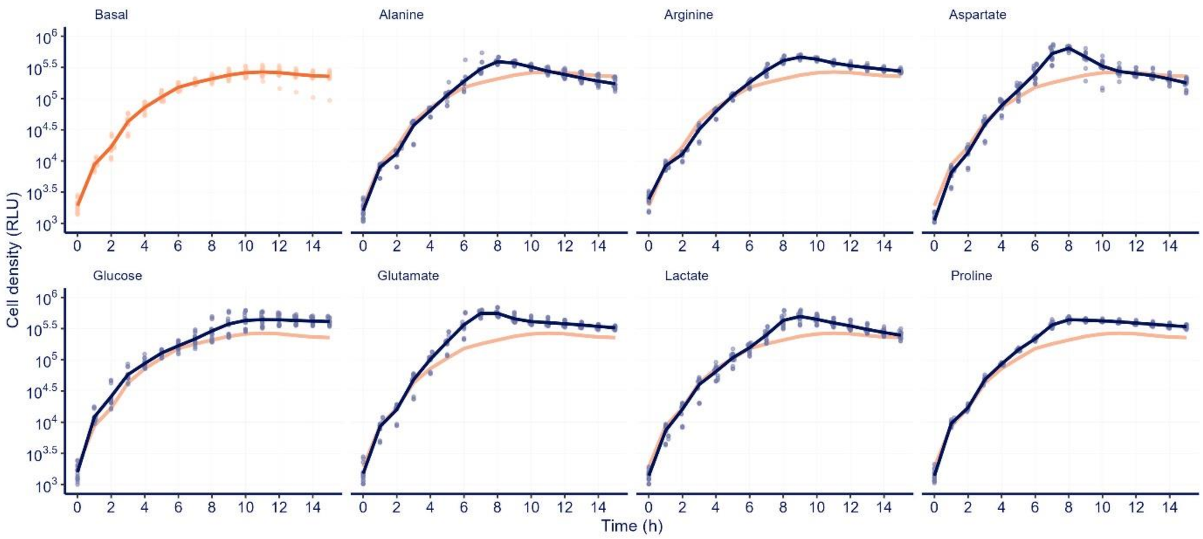

Supplemental figure 4. The dynamic effect of the addition of nutrients (navy blue lines) to the basal (orange) media composition on the population size over 15-hours of incubation in antibiotic free conditions.

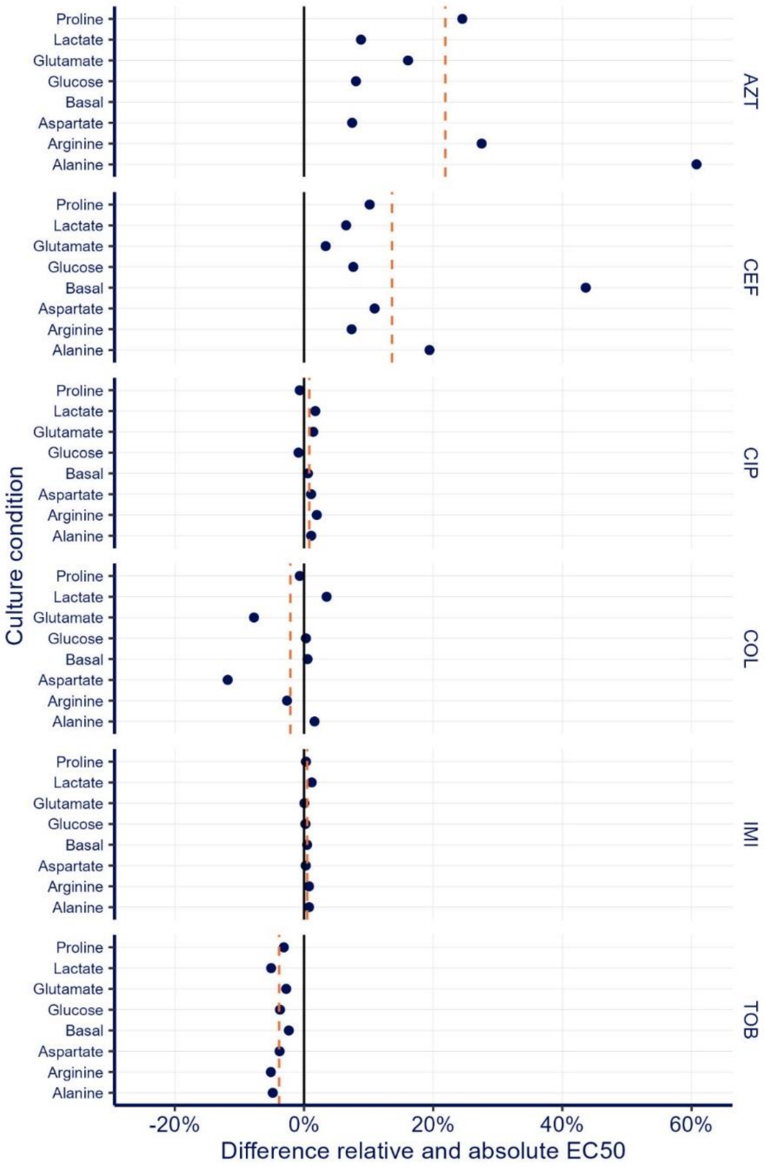

Supplemental figure 5. The difference between the relative half-maximal effective concentration (EC_{50}) and the absolute EC_{50}. The relative EC_{50} is extracted as the halve maximal response of the dose-response curve between the population fitness (E_0) and the maximal drug effect (E_{max}). The absolute EC_{50} is extracted as the concentration at 50% reduction of E_0. The difference between the two antibiotic sensitivity determinations is obtained by dividing the relative EC_{50} by the absolute IC_{50} concentrations per culture condition. *Abbreviations: aztreonam (AZT), ceftazidime (CAZ), ciprofloxacin (CIP), colistin (COL), imipenem (IMI), and tobramycin (TOB).*

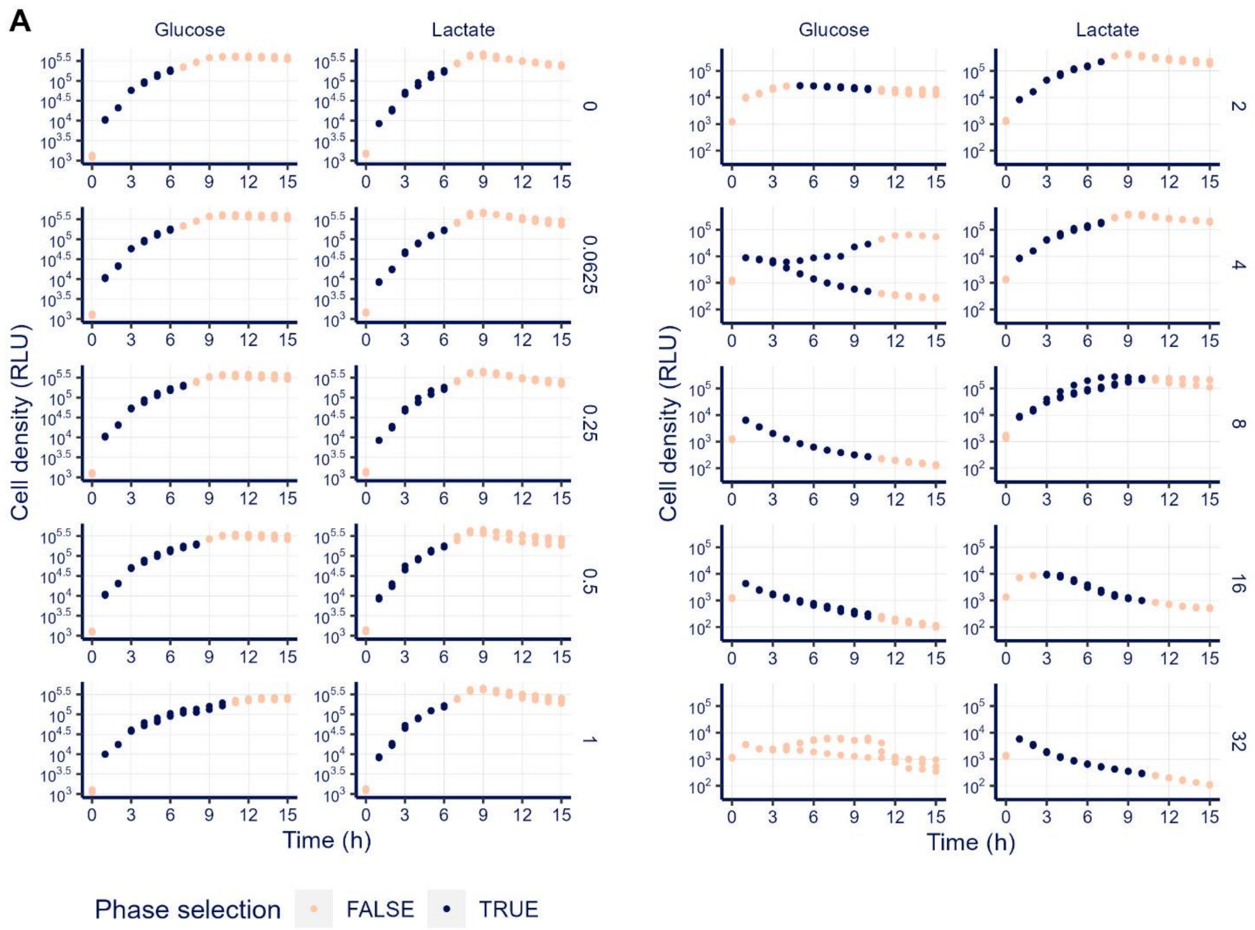

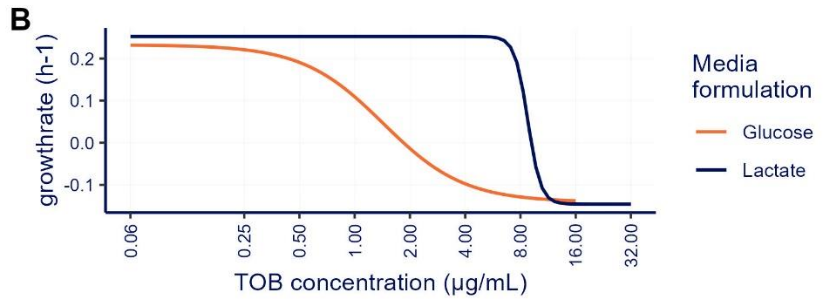

C

Parameter	Glucose	Lactate
EC50	1.41	8.58
μmax	0.24	0.25
Emax	-0.14	-0.15
Hill	1.85	16.06

Supplemental figure 6. Phase selection for growth rate determination for growth rate based dose-response modeling of tobramycin (TOB). (A) The time-points included (blue dots) for the determination of the growth or kill rate of the tobramycin concentration using a linear regression. (B) The sigmoid E_{max} dose-response curve for glucose and lactate using the growth rate as response. (C) The pharmacodynamic parameters extracted from the dose-response model.

Supplemental table 1. Detailed content list of synthetic media

	Name	Concentration (mM)	Company information
M9 buffer	di-sodium hydrogen phosphate (Na_2HPO_4)	90.2	Thermo Fisher Scientific , Breda, The Netherlands
	Potassium di-hydrogen phosphate (KH_2PO_4)	22.0	VWR International, Amsterdam, The Netherlands
	Sodium chloride (NaCl)	8.5	Merck KGaA (Avantor™), Darmstadt, Germany
	Ammonium chloride (NH_4Cl)	18.6	Alfa Aesar (Thermo Fisher GmbH), Kandel, Germany
	Magnesium sulphate hepta-hydrate ($MgSO_4$)	1.0	VWR International, Amsterdam, The Netherlands
	Calcium chloride ($CaCl_2$)	0.1	Acros Organics (Thermo Fisher Scientific), Geel, Belgium
Salts	Potassium nitrate (KNO_3)	0.35	Acros Organics (Thermo Fisher Scientific), Geel, Belgium
	Iron sulphate ($FeSO_4$)	0.0036	Alfa Aesar (Thermo Fisher GmbH), Kandel, Germany
Vitamins	BME Vitamin solution	*1x*	Thermo Fisher Scientific , Breda, The Netherlands
Trace metals	Di-sodium Ethylene di-amine tetra-acetic acid (EDTA)	0.002 (mg/mL)	J.T. Baker (Avantor™), Darmstadt, Germany
	Zinc Sulphate hepta-hydrate ($ZnSO_4$)	0.23 (mg/mL)	Alfa Aesar (Thermo Fisher GmbH), Kandel, Germany
	Boric acid (H_3BO_3)	0.111 (mg/mL)	Acros Organics (Thermo Fisher Scientific), Geel, Belgium
	Manganese chloride tetra-hydrate ($MnCl_2$)	0.051 (mg/mL)	Sigma Aldrich (Avantor™), Darmstadt, Germany
	Cobalt chloride ($CoCl_2$)	0.017 (mg/mL)	Alfa Aesar (Thermo Fisher GmbH), Kandel, Germany
	Copper Sulphate penta-hydrate ($CuSO_4$)	0.015 (mg/mL)	Sigma Aldrich (Avantor™), Darmstadt, Germany
	Ammonium hepta-molybdate tetra hydrate ($(NH_4)_6\ Mo_7O_2$)	0.01 (mg/mL)	Alfa Aesar (Thermo Fisher GmbH), Kandel, Germany
Basis nutrients	Cysteine (Cys)	0.2	Chem-Impex International, Wood Dale, IL, USA
	Glycine (Gly)	1.2	Acros Organics (Thermo Fisher Scientific), Geel, Belgium
	Histidine hydrochloride (His)	0.5	Chem-Impex International, Wood Dale, IL, USA
	Isoleucine (Ile)	1.1	Chem-Impex International, Wood Dale, IL, USA
	Leucine (Leu)	1.6	Chem-Impex International, Wood Dale, IL, USA
	Lysine hydrochloride (Lys)	2.1	Thermo Fisher Scientific , Breda, The Netherlands
	Methionine (Met)	0.6	Chem-Impex International, Wood Dale, IL, USA
	Phenylalanine (Phe)	0.5	Chem-Impex International, Wood Dale, IL, USA
	Serine (Ser)	1.4	Chem-Impex International, Wood Dale, IL, USA
	Threonine (Thr)	1.0	Chem-Impex International, Wood Dale, IL, USA
	Tryptophan (Trp)	0.01	Chem-Impex International, Wood Dale, IL, USA
	Tyrosine (Tyr)	0.8	Chem-Impex International, Wood Dale, IL, USA
	Valine (Val)	1.1	Chem-Impex International, Wood Dale, IL, USA
Nutrient alterations	Alanine (Ala)	15	Chem-Impex International, Wood Dale, IL, USA
	Arginine (Arg)	15	Chem-Impex International, Wood Dale, IL, USA
	Aspartate (Asp)	15	Chem-Impex International, Wood Dale, IL, USA
	Glutamate (Glu)	15	Chem-Impex International, Wood Dale, IL, USA
	Sodium lactate (LAC)	15	Biosynth International, Compton, United Kingdom
	Proline (Pro)	15	Thermo Fisher Scientific , Breda, The Netherlands
	Glucose (GLC)	15	Alfa Aesar (Thermo Fisher GmbH), Kandel, Germany

Supplemental table 2. Pharmacokinetic parameters

Explanation	Name	Value / Formula	Unit
Patient bodyweight	BW	55.3	kg
Patient age		29.0	years
Clearance rate per BW	CL_t	0.1212	L/h/kg
Volume comp. 1 per BW	V_C	0.20	L/kg
Distribution rate per BW	CL_d	0.0702	L/h/kg
Volume comp. 2 per BW	V_{ss}	0.38	L/kg
Individual Variability (η)	η_{CL_t}<br>η_{V_C}<br>η_{CL_d}<br>$\eta_{V_{ss}}$	28.5<br>28.2<br>66.6<br>27.8	%<br>%<br>%<br>%
Population size		1000	
Dosing interval		8	h
Dosing amount		3.3 * BW	mg
Dosing duration		0.30	h
Volume compartment 1	$V_{central}$	$V_C * e^{\{iv\}} * BW$	L
Elimination rate from $V_{central}$	$k_{elimination}$	$(CL_t * e^{\{iv\}} * BW) / V_{central}$	
Volume compartment 2	V_2	$V_{ss} * e^{\{iv\}} * BW$	L
Rate constant 1-->2	K_{12}	$(CL_d * e^{\{iv\}} * BW) / V_{central}$	
Rate constant 2-->1	K_{21}	$(CL_d * e^{\{iv\}} * BW) / V_2$	
Amount in compartment 1	$m_{central}$	$\frac{m_{central}(t)}{dt} = k_{21} * m_2 - (k_{elimination} + k_{12}) * m_{central}$	mg
Amount in compartment 2	m_2	$\frac{m_2(t)}{dt} = K_{12} * m_{central}(t) - k_{21} * m_2(t)$	mg
Concentration compartment 1	$C_{central}$	$m_{central} / V_{central}$	mg/L

Supplemental table 3. Pharmacodynamic parameters

Explanations	Name	Value / Formula		Unit
		Glucose	Lactate	
Max. drug effect	E_{max}	-0.144	-0.146	$^{-}$h
Max. growth rate	K_{growth} (E_0)	0.240	0.254	$^{-}$h
Half effective concentration	EC_{50}	1.406	8.582	mg/L
Hill coefficicent	n_H	1.850	16.057	
Starting population	N_0	$1 * 10^6$		CFU/mL
Max. population	N_{max}	$9 * 10^9$		CFU/mL
Effective growth rate	k_{effect}	$K_{growth} - (E_{max} + \frac{K_{growth} - E_{max}}{1 + e^{n_H(\log(C_{central}) - \log(EC_{50}))}})$		$^{-}$h
Infection population	N(t)	$\frac{dN(t)}{dt} = (k_{growth} * (1 - N(t)/N_{max}) - k_{effect}) * N(t)$		CFU/mL
